# Polyhydroxyalkanoates in Bone Alloplastic Materials: State of the Art and Future Perspectives

**DOI:** 10.3390/polym18121508

**Published:** 2026-06-16

**Authors:** Alessandro Mosca Balma, Sara Meinardi, Ilaria Roato, Federico Mussano

**Affiliations:** 1 Bone and Dental Bioengineering Laboratory, CIR Dental School, Department of Surgical Sciences, University of Turin, via Nizza 230, 10126 Turin, Italy; alessandro.moscabalma@unito.it (A.M.B.); or sara.meinardi@polito.it (S.M.); ilaria.roato@unito.it (I.R.); 2 Department of Mechanical and Aerospace Engineering, Politecnico di Torino, Corso Duca degli Abruzzi 24, 10129 Turin, Italy; 3 Department of Life Sciences and Systems Biology, University of Turin, via Accademia Albertina 13, 10123 Turin, Italy

**Keywords:** polyhydroxyalkanoates, bone substitutes, bio-based polymers, additive manufacturing, mechanical properties, biological properties, bone tissue engineering

## Abstract

Polyhydroxyalkanoates (PHAs) are bio-based, biodegradable polyesters increasingly explored as sustainable biomaterials for regenerative medicine. This review summarizes recent advances in PHA-based bone substitute materials, highlighting their properties, fabrication methods, and biological performance. PHAs combine biocompatibility, tunable mechanical behavior, and degradation into non-toxic metabolites, while copolymerization and monomer selection modulate the stiffness, crystallinity, and resorption rate. Processing techniques such as solvent casting, electrospinning, and additive manufacturing allow the production of porous architectures that mimic bone extracellular matrix. Electrospinning is particularly suitable for nanoscale fibrous matrices, whereas 3D printing enables patient-specific scaffolds with controlled geometry and interconnected porosity. Scaffold performance can be further improved through the incorporation of osteoconductive fillers, including hydroxyapatite, β-tricalcium phosphate, bioactive glasses, graphene oxide, and carbon nanotubes, as well as through drug-delivery and pro-angiogenic functionalization. In vitro and in vivo studies consistently report favorable cytocompatibility, enhanced osteogenic differentiation, vascularization, and effective repair of bone defects in animal models. However, clinical translation remains limited by production costs, variability in polymer quality, thermal processing constraints, and regulatory challenges. Future progress will rely on more efficient biosynthesis, medical-grade purification, multifunctional scaffold design, and stronger collaboration between academia, industry, and clinicians to unlock the full potential of PHAs in regenerative bone therapies.

## 1. Introduction

The development of artificial scaffolds for bone replacement (alloplasts) that mimic the composition and architecture of the osseous extracellular matrix plays a fundamental role in the field of bone tissue engineering (BTE) [1,2,3]. Artificial scaffolds may offer advantages compared to cadaveric allografts and animal-derived xenografts, which are hindered by reduced biocompatibility, more challenging graft integration, and a potential risk of viral or bacterial transmission [2,3]. Furthermore, alloplasts avoid the restricted donor site availability and the risk of donor site morbidity proper of autologous grafts [4].

Biocompatibility, open and interconnected porosity, mechanical properties appropriate to the host tissue, and bioactivity are among the key characteristics required for materials intended for bone grafting [5]. Thus, an ideal bone scaffold should provide both structural support at the defect site and a suitable physiological environment to guide osteoblastic adhesion, proliferation, and subsequent bone formation [2,3]. Furthermore, alloplastic grafts may be functionalized as delivery systems for bioactive molecules, including growth factors and therapeutic agents, thereby enhancing their regenerative potential and broadening their clinical applications [6,7].

Alloplasts are usually composite materials made of biodegradable polymers, like polylactic acid (PLA), poly(lactide-co-glycolide) (PLGA), polycaprolactone (PCL), and collagen composites, charged with ceramic fillers mimicking the bone mineral composition such as β-TCP or carbonated hydroxyapatite (HA) [8]. Due to molding properties that facilitate tridimensional printing, PCL, a synthetic and bioresorbable aliphatic polyester, has become the benchmark among the printable polymers for BTE applications [9,10,11,12,13,14,15]. Nevertheless, its petroleum-based origin is associated with negative ecological and environmental implications [16].

Consequently, renewable resources for polymer production have been increasingly explored by both academic and industrial researchers. Polyhydroxyalkanoates (PHAs), a family of polyesters produced by fermentation from natural resources (i.e., sugar or lipids), have emerged owing to their favorable biocompatibility and biodegradability [17,18]. Following the first anecdotal observation by Martinus W. Beijerinck in 1888 and the pioneering work of Maurice Lemoigne, who identified and characterized poly(3-hydroxybutyrate) (PHB) in the early twentieth century [19], more than 150 distinct PHA monomers have been described, making PHAs the largest class of natural polyesters [20].

A key milestone supporting the commercial relevance of PHAs and their emergence as bioplastics was the early demonstration of the biodegradability and biocompatibility of PHB [21], together with the recognition that its properties are comparable to those of polypropylene [22]. These favorable features were further confirmed by the absence of toxic degradation products and carcinogenic effects in other PHAs [23], exhibiting physicochemical properties similar to conventional synthetic polymers [24]. As they also possess favorable processability and can be fabricated into materials with diverse architectures and morphologies using salt-leaching and solution casting [25,26,27], electrospinning [28], and other additive manufacturing techniques [18,29], PHAs are now considered promising candidates for tissue engineering. Their appeal is further strengthened by reported antibacterial and antioxidative properties [30].

This review aims to consolidate recent advancements and provide critical insights into the application of PHAs as bone alloplastic materials. It presents a comprehensive overview of the current literature, followed by a discussion of future research directions in this field.

## 2. Classification of PHAs

**Figure 1:**
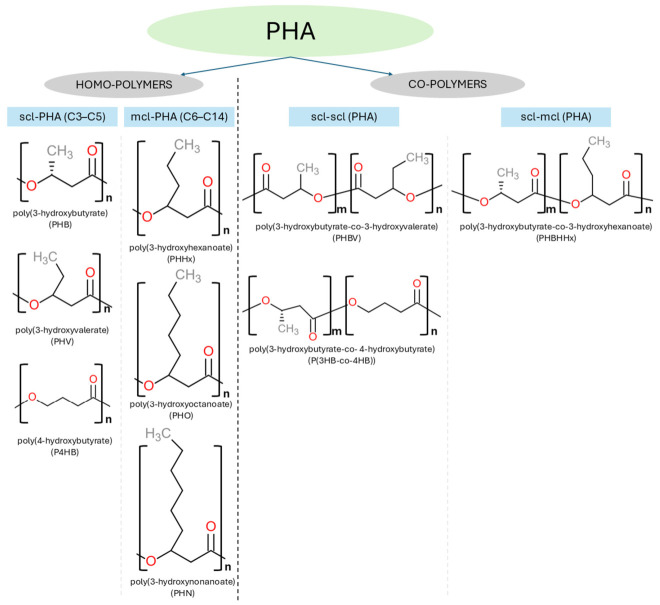
Classification of most widely discussed PHA categories into homopolymers and co-polymers. mcl-PHA: medium-chain-length polyhydroxyalkanoate; scl-PHA: short-chain-length polyhydroxyalkanoates.

PHAs comprise a diverse group of biopolymers primarily composed of 3-hydroxyacids. PHAs are classified based on monomer chain length (Figure 1) into short-chain-length (scl-PHAs, C3–C5) and medium-chain-length PHAs (mcl-PHAs, C6–C14) [20], and long-chain-length PHAs (lcl-PHAs, C > 15). To date, more than 150 different monomeric units have been identified [31], enabling the production of PHAs with a broad range of physicochemical and mechanical properties. Chain length distribution critically determines physicochemical properties, including crystallinity, melting temperature (T_m_), glass transition temperature (Tg), tensile strength, and elongation at break. Tg generally ranges from −35 °C to 10 °C, while T_m_ ranges from 49 °C to 177 °C [32]. scl-PHAs are typically highly crystalline (>60%) and brittle, with melting points ranging from 150 to 180 °C [33], whereas mcl-PHAs exhibit lower crystallinity (~20–30%), reduced T_m_ (42–65 °C), and elastomeric behavior due to increased chain mobility and reduced intermolecular packing [34]. Importantly, the modulation of PHA composition allows tuning of both Tg and T_m_ values. For example, the incorporation of 25% 3-hydroxyvalerate (3-HV) units into PHB decreases the melting temperature from 177 °C to 137 °C. However, the overall degree of crystallinity remains substantially unchanged, as the two monomeric units are capable of co-crystallization [31]. This makes poly(3-hydroxyhexanoate) (PHHx), poly(3-hydroxyoctanoate) (PHO), and poly(3-hydroxynonanoate) (PHN) suitable for applications demanding more pliable materials, while poly(3-hydroxybutyrate) (PHB), the simplest of PHAs, belonging to scl-PHAs, is the most rigid PHA, with limited processability and a rapid aging rate.

PHAs can be synthesized as homopolymers, consisting of a single monomer unit, or as copolymers incorporating two or more monomers along the polymer chain. Copolymers such as poly(3-hydroxybutyrate-co-3-hydroxyvalerate) (PHBV) and poly(3-hydroxybutyrate-co-3-hydroxyhexanoate) (PHBHHx) exhibit tunable properties, thereby broadening their applicability across diverse fields [35]. The most relevant PHAs for biomedical applications are PHB, poly(3-hydroxyvalerate) (PHV), their copolymer PHBV, PHHx, and PHO. PHB and some of its copolymers are available in medical grade form [36,37]. For a comprehensive overview of the physicochemical and mechanical properties of the different PHAs, please refer to the review by Mehrpouya et al. [31].

## 3. Production, Extraction and Purification of PHA

PHAs are intracellular carbon and energy storage polymers synthesized by microorganisms in response to environmentally stressful conditions [35]. Since the discovery of PHB accumulation in *Bacillus megaterium*, numerous PHA-producing bacterial strains have been identified across a wide range of ecological niches. To date, hundreds of pure-culture microorganisms (PCMs) have been reported as natural PHA producers [38], including a variety of genetically engineered strains designed to enhance productivity or tailor polymer properties [39,40]. While PCMs generally enable high yields and well-defined product composition, increasing attention has been directed, since the early 2000s, toward mixed microbial cultures (MMCs) as a cost-effective alternative. MMC-based systems can exploit inexpensive and renewable feedstocks, such as organic waste and wastewater streams [41,42,43,44]. However, MMCs typically exhibit lower intracellular PHA accumulation due to microbial diversity and interspecies competition (Table 1).

In recent years, research has expanded significantly, with particular emphasis on the discovery of novel PHA-producing organisms, the development of engineered strains with improved yields or tailored polymer composition, and the advancement of pilot-scale production processes alongside material characterization and application studies [71].

Among the bacteria used for industrial-scale PHA production, *Pseudomonas putida* KT2440 [72] and *Pseudomonas oleovorans* ATCC 29347 [73] are well-known producers of mcl-PHAs. In addition, *Burkholderia sacchari* [74] and *Cupriavidus necator* H16 are widely employed for PHB synthesis, with the latter capable of utilizing a broad range of carbon substrates and achieving high yields [75]. The use of halophilic microorganisms, such as *Haloferax mediterranei*, represents a promising strategy to reduce production costs, as the high-salinity conditions required for their growth inhibit contamination, thereby minimizing the need for sterile operation [76]. In summary, PHA production is strongly influenced by multiple factors, including the carbon-to-nutrient ratio, the microbial strain employed, and the specific cultivation conditions.

Extracting PHA from microbial biomass is a key downstream process that determines both polymer purity and process economics. Mcl-PHAs can be efficiently recovered using relatively mild solvents (e.g., acetone, ethyl acetate), whereas scl-PHAs, like PHB, are typically extracted with halogenated solvents (e.g., chloroform), achieving high purity (86–99%) and recovery yields up to 94% [77]. However, toxicity and environmental concerns significantly limit their industrial applicability. Consequently, non-halogenated alternatives, including anisole, phenetole, and cyclohexanone, have been investigated, showing promising PHB recovery performance [78].

Digestion-based methods, which selectively remove non-PHA biomass while preserving intracellular granules, represent a widely explored strategy. Early approaches employing sodium hypochlorite or sodium hydroxide achieved recovery yields up to 90%, albeit with risks of polymer degradation under harsh conditions [79]. Mechanical disruption techniques, such as bead milling and high-pressure homogenization, eliminate solvent use but remain energy-intensive and challenging to scale [80]. More advanced methods, including supercritical carbon dioxide extraction, enzymatic digestion, solvent-based gelation, ultrasound-assisted extraction, and ionic liquid systems, offer improved sustainability profiles, though often at the expense of higher cost and process complexity.

Overall, the physicochemical properties of PHAs, such as crystallinity, stiffness, elasticity, and degradation rate, can be finely tuned through monomer composition, strain selection, substrate type, fermentation conditions, and downstream processing, including extraction. This tunability has driven the development of engineered strains and advanced bioprocessing strategies, enabling the production of tailored PHA materials for specific applications [35].

Moreover, producing medical-grade PHAs requires efficient purification to remove residual pyrogens, namely endotoxins and exotoxins derived from microbial biomass, using mechanical or chemical approaches. For instance, filtration of polymer solutions through activated charcoal can effectively reduce endotoxin levels without significant polymer degradation [81]. Alternatively, oxidative treatments, such as hydrogen peroxide or potassium permanganate, may be employed, preserving polymer integrity [82]. In addition, ethylene oxide treatment and gamma irradiation can simultaneously ensure sterilization and depyrogenation [83].

To date, PHAs have been effectively used to produce a series of biomedical devices. Notably, TephaFLEX^®^, an absorbable surgical suture based on poly(4-hydroxybutyrate) (P(4HB)), was the first PHA-based material approved by the U.S. Food and Drug Administration for clinical use in 2007. Additional commercial PHA-based products include GalaFLEX^®^ and Phasix™ Mesh for reconstructive and plastic surgery, BioFiber^®^ for tendon repair, and MonoMax^®^ and Phantom Fiber^®^ sutures [71]. However, despite these advances, the application of PHAs in BTE has not yet achieved clinical translation.

Several factors contribute to this limitation. While sutures and meshes are classified as Class II medical devices and therefore undergo a less stringent regulatory clearance pathway, bone scaffolds, due to longer residence times and structural complexity, frequently require the more rigorous premarket approval (PMA) process. Accordingly, long-term degradation studies must assess systemic toxicity, chronic inflammatory responses, and tissue sensitization associated with localized degradation products, in compliance with the ISO 10993 biocompatibility requirements [84].

Sterilization validation also represents a major challenge. Common sterilization methods, such as gamma irradiation, may induce polymer chain scission, substantially altering the molecular weight, degradation kinetics, and mechanical integrity of PHA-based materials. While such effects may be less critical for sutures, they can severely compromise the mechanical performance of bone scaffolds.

Finally, batch-to-batch consistency remains a crucial issue. Due to their bacterial origin, PHAs are intrinsically subject to biological variability. Therefore, robust quality control procedures are essential to ensure reproducible molecular weight distributions and consistent mechanical properties, both of which are fundamental requirements for bone alloplastic materials.

## 4. Solvent Casting Methods for PHA-Based Scaffolds

The group of Prof. Boccaccini was among the first to propose PHAs for BTE scaffolds. In 2009, they reported the incorporation of vitamin E in PHB/bioglass (BG) composite films [85] and the implementation of PHB/BG foams with high interconnected porosity using a novel combination of solvent casting and particulate leaching techniques [86]. The foams allowed MG-63 cell attachment and proliferation while exhibiting bactericidal properties as assessed on the growth of *Staphylococcus aureus*. A non-toxic foreign body response occurred after one week of subcutaneous implantation in rats. Moreover, multifunctional PHB/BG constructs incorporating selected concentrations of vitamin E or/and carbon nanotubes were produced, paving the way for next generation BTE scaffolds.

Wang et al. [87] recurred to the solvent casting-particulates leaching method to fabricate β-dicalcium silicate/PHBV composites, which compared to neat PHBV scaffolds, facilitated the adhesion of MG-63 cells owing to their increased hydrophilicity and enhanced their osteogenic potential. Similarly, PHBV scaffolds were loaded with icariin, improving the proliferation of murine pre-osteoblasts (MC3T3-E1) and human MG-63 cells [88].

When analyzing in vitro models of osteogenic potential, it should be noted that osteosarcoma-derived cell lines, such as MG-63 and SaOS-2, have been widely used, particularly in earlier studies, due to their ease of handling and relatively stable osteogenic differentiation capacity [89]. However, by definition, immortalized tumor-derived cells are characterized by altered chromosomal profiles and therefore may not faithfully reproduce the complex regulatory mechanisms, gene expression patterns, or physiological mineralization behavior of primary osteoblasts or mesenchymal stem cells (MSCs).

For example, MG-63 cells exhibit an immature pre-osteoblastic phenotype, characterized by basal alkaline phosphatase (ALP) activity and mineralization rates significantly lower than those observed in primary human osteoblasts, together with almost absent osteocalcin production, a key marker of late-stage osteogenesis [90,91]. Consequently, the use of MSCs is currently considered preferable to osteosarcoma-derived cell lines so as to reproduce, as closely as possible, the physiological bone microenvironment.

## 5. Electrospun Materials Mimicking Bone Extracellular Matrix and Promoting Vascularization

The complexity of bone matrix architecture can be more effectively mimicked using advanced fabrication strategies capable of integrating multiple materials and functional cues. Recently, dual-nozzle hybrid electrospinning has enabled the simultaneous processing of polymers from distinct solvent systems. A resume of the main electrospun materials is reported in Table 2 and discussed below. Using this approach, poly(3-hydroxybutyric acid-co-4-hydroxybutyric acid) (P34HB) and polyvinyl alcohol (PVA) were combined with multi-walled carbon nanotubes (MWCNTs), yielding hierarchical constructs with tunable properties [92]. In vitro assays on human bone marrow stromal cells (BMSCs) confirmed cytocompatibility and osteogenic potential, while in vivo implantation, in a rabbit femur model, demonstrated improved bone integration and screw stability, particularly for scaffolds containing 1% MWCNTs. Earlier, electrospun PHB/starch composites loaded with 1 wt% MWCNTs showed enhanced mechanical performance (tensile strength: 24.37 ± 0.22 MPa) and promising osteoconductive properties in MG-63 cells [93].

A biomimetic approach was further demonstrated by Sriram et al., who developed electrospun multi-interface scaffolds designed to replicate osteon hierarchy [94]. In this system, PHB/gelatin fibers promoted anisotropic elongation and mineralization of mouse BMSCs, whereas PHB/polypyrrole fibers supported neural cell growth, enabling the design of bi-layered constructs potentially suitable for recreating Haversian canal-like structures with integrated vascular and neural components.

Early efforts in electrospun PHA-based composites include PHBV systems reinforced with HA, which consistently demonstrated improved osteoconductivity. Electrospun PHBV containing natural poly(α,β)-DL-aspartic acid and HA nanofibers enhanced proliferation and mineral deposition in human fetal osteoblasts (hFOBs) compared to neat PHBV [116]. Similarly, HA nanoparticle incorporation into PHB scaffolds improved in vitro biocompatibility and osteogenic response [117], while in vivo experiments in BALB/c nude mice confirmed osteoid formation and vascular ingrowth after 2 months [112]. To improve vascularization in bone tissue engineering, electrospun PHBV fiber meshes have also shown promising results when combined with endothelial-differentiated cells [96]. PHB-HV scaffolds seeded with hASCs did not significantly enhance bone regeneration; however, they promoted vascularization in a nude mouse model of critical-sized calvarial defects [119]. Furthermore, poly(3-hydroxybutyrate-co-4-hydroxybutyrate) (P34HB) fibrous wound dressings loaded with the antibacterial agent ciprofloxacin (CIP) and the pro-angiogenic molecule dimethyloxalylglycine (DMOG) effectively promoted capillary-like tube formation while simultaneously exerting antimicrobial activity in a skin wound-healing model [97].

Further developments include PHBV/SiHA electrospun scaffolds, which outperformed PHBV and PCL in terms of adhesion, proliferation, and osteogenic differentiation of human mesenchymal stromal cells (MSCs) [111], as well as PHBV/polyaspartic acid systems with in situ HA formation, which enhanced osteogenic activity in hFOB cells [113]. Additional reinforcement strategies involved silk fibroin (SF), which improved early cell response and osteogenic differentiation in PHB-based nanofibers [115,118] and non-woven electrospun films composed of a PHBHHx [105]. N_2_ plasma treatment of electrospun PHBV fibers prior to SF adsorption improved Saos-2 cell viability [114], while atmospheric plasma treatment further enhanced PHB nanofiber wettability, reducing the water contact angle from ~120° to ~43° and improving cellular response [100].

Other reinforcing phases have also been explored. Alumina nanowires significantly improved mechanical strength (>10-fold increase in tensile properties) and enhanced MG-63 cell proliferation in PHB/chitosan systems [108], while PHB/keratin composites reinforced with alumina (3 wt%) exhibited a tensile strength of ~6.72 MPa and osteoinductive potential [99]. Graphene oxide (GO) incorporation improved hydrophilicity, mechanical properties, and mineralization in PHB/chitosan scaffolds without compromising cytocompatibility [98], and GO-containing P34HB scaffolds demonstrated successful bone regeneration in rat calvarial defects [110].

Biological and bioactive fillers have further expanded scaffold functionality. Diatom shells introduced silicon-based bioactivity and improved Saos-2 cell viability in PHBV/PCL systems [106], while aloe vera-derived gels enhanced proliferation and osteogenic differentiation of human-induced pluripotent stem cells (iPSCs) in PHBV scaffolds [101]. Zein-loaded PHB scaffolds also exhibited improved osteoconductivity in MG-63 cells [95], while adding fish (*Sparus aurata*) scales to PHBV enhanced the compressive strength of the composite material, biomineralization in simulated body fluid, cell proliferation, alkaline phosphatase activity, and type I collagen production in MG-63 cells [104].

Advanced composite membranes combining PHBV with fibrinogen and bredigite enhanced mechanical and osteogenic performance for guided bone regeneration (GBR), with increased ALP activity and mineralization in hFOB cells [109]. Similarly, P34HB/octacalcium phosphate (OCP) nanofibers promoted MSC differentiation and de novo bone formation in vivo [103]. P34HB woven electrospun scaffolds also supported calvarial defect repair in rats [107].

Finally, blending PHBV with poly(l-lactide) (PLLA) via electrospinning addressed the brittleness limitations of PLLA. The resulting ultrafine composite fibers exhibited tunable thermal and mechanical properties, with PHBV reducing Tg and increasing the Young’s modulus. The PLLA/PHBV 70/30 formulation showed improved shape memory behavior and osteoinductive effects in mouse MSCs [102].

## 6. 3D Printing Technologies for PHA-Based BTE Scaffolds

Additive manufacturing (AM) has emerged as a fast, reliable, and highly versatile approach for processing biomaterials with complex geometries [31] (Table 3). Early translational evidence was provided by Berger et al., who in 2015 demonstrated the technical and cell biological feasibility of BTE for alveolar cleft osteoplasty [120]. Based on cone-beam CT reconstructions of patient-specific alveolar defects, custom tricalcium phosphate (TCP) scaffolds were fabricated, sintered, and subsequently infiltrated with PHB. Biocompatibility was assessed using human MSCs via the WST-1 assay and scanning electron microscopy (SEM) analysis, while osteogenic differentiation was evaluated through alkaline phosphatase (ALP) activity. The high geometric fidelity of the constructs and encouraging in vitro performance highlighted the potential of patient-specific AM scaffolds, warranting further progression toward clinical translation. Selective laser sintering (SLS) allowed for the printing of hierarchical PHB porous scaffolds with favorable mechanical properties [121]. Functionalization with osteogenic growth peptide (OGP) and its C-terminal fragment OGP(10–14) further supported the proliferation and osteogenic differentiation of rat BMSCs, while good cell viability was maintained across all PHB scaffold formulations.

Subsequent studies have extensively explored 3D-printed PHAs for BTE applications, including fused deposition modeling (FDM) approaches [134]. A notable early example is the work by Yang et al., who developed composite scaffolds based on PHBHHx using combined 3D printing and surface modification with mesoporous BG [133]. Morphological and structural analyses confirmed homogeneous BG coating throughout the porous architecture. These hierarchical constructs exhibited enhanced bioactivity compared to neat PHBHHx, with improved human MSC adhesion, proliferation, and osteogenic differentiation attributed to the BG-derived ionic environment.

More recently, melt-extrusion 3D printing has been used to fabricate scaffolds based on mcl-PHAs, specifically poly(3-hydroxyoctanoate-co-hydroxydecanoate-co-hydroxydodecanoate) [126]. These constructs showed excellent cytocompatibility, with 100% viability in both undifferentiated and differentiated MC3T3-E1 cells. Antibacterial functionality was introduced via two strategies: blending with sulfur-containing PHAs (PHACOS), which reduced *Staphylococcus aureus* contamination by ~70%, and incorporation of selenium- and strontium-doped hydroxyapatite (Se-Sr-HA, 10 wt%), which conferred broad-spectrum antibacterial activity against both S. aureus and *Escherichia coli* without compromising osteoblastic viability.

To impart angiogenic cues, dimethyloxallyl glycine (DMOG), a prolyl-4-hydroxylase inhibitor that stabilizes hypoxia-inducible factor-1α (HIF-1α), was incorporated into scaffolds composed of mesoporous bioactive glasses and PHBHHx [131]. In vitro assays on human BMSCs demonstrated enhanced early biological responses, together with promoted osteogenic and angiogenic differentiation. In vivo evaluation in rat calvarial defects (12-week-old Sprague-Dawley rats) showed significantly increased vascularization and larger areas of new bone formation in DMOG-loaded scaffolds compared to the controls. In a related approach, 3D-printed PHBHHx scaffolds were also loaded with therapeutic agents for the treatment of bone tuberculosis [132]. In a New Zealand rabbit femoral defect model, these drug-loaded constructs exhibited satisfactory osteogenic performance, highlighting their potential as multifunctional platforms combining structural support and localized drug delivery.

The role of β-TCP in improving the biological and mechanical performance of the proprietary PHA Ecomann^®^ Bioresin EM10080, originally developed for biofilm applications, was investigated by Ye et al. [18]. FDM-fabricated composites containing 0–30 wt% β-TCP were evaluated, with the 20 wt% formulation showing the best mechanical performance. In particular, the PHA/20% β-TCP scaffold exhibited a compressive strength of 36.7 MPa and a Shore hardness of 81.1 HD. In vitro assays demonstrated that β-TCP significantly enhanced MC3T3-E1 cell proliferation, adhesion, and migration. Moreover, the expression of osteogenesis-related genes was markedly upregulated compared with neat PHA scaffolds.

The same FDM-processed Ecomann^®^/β-TCP composite was later functionalized with copper-containing carboxymethyl chitosan/alginate (CA/Cu) hydrogels [128]. This multifunctional scaffold further promoted pre-osteoblast proliferation and osteogenic differentiation, while providing strong broad-spectrum antibacterial activity, including against methicillin-resistant *Staphylococcus aureus* (MRSA). In vivo studies further demonstrated enhanced repair of cranial defects together with effective eradication of MRSA-associated infection, thereby addressing a major clinical challenge in bone regeneration.

Claiming improved biocompatibility, Domínguez-Candela et al. incorporated selected additives into PHB/PLA blends processed by material extrusion (MEX) [125]. The compatibilizers included petroleum-derived poly(ethylene) with glycidyl methacrylate (EGM), methyl acrylate-co-glycidyl methacrylate (EMAG), poly(styrene-co-maleic anhydride) (Xibond), and the bio-based additive epoxidized linseed oil (ELO). These additives enhanced phase compatibility within the PHB/PLA blends, with EMAG and ELO showing the most pronounced improvement in ductility. Processability was also improved through reduced melt temperature and enhanced thermal stability. The resulting scaffolds exhibited compressive strengths of 11–13 MPa, exceeding the typical range reported for trabecular bone (5–10 MPa). The highest cell metabolic activity was observed for formulations containing ELO and Xibond, likely due to the reduced water contact angle. SEM analysis further confirmed stable cell attachment after 7 days of culture.

One of the most notable recent advances was the development of a fully resorbable PHBV scaffold evaluated both in vitro and in vivo, providing strong evidence that PHA-based systems can function as bone substitutes even in the absence of bioactive fillers [127]. Mechanical testing showed that the 3D-printed scaffolds achieved a maximum Young’s modulus of 241.34 ± 7.62 MPa and a compressive strength of 22.43 ± 1.89 MPa. SEM observations of adherent cells and MTT assays on NIH 3T3 fibroblasts confirmed good cytocompatibility for up to 10 days of culture. The osteoconductive potential of the constructs was further validated in a femoral diaphyseal defect model in domestic Landrace pigs, with mature bone formation and complete defect restoration after 150 days.

A particularly noteworthy example is the extrusion-based 3D-printed PHB scaffold functionalized with dextran and charged Mg-doped whitlockite nanoparticles, further integrated with a sildenafil-releasing drug delivery system intended to provide pro-angiogenic cues [122]. The scaffold showed a compressive strength of 3.70 ± 0.33 MPa and an elastic modulus of 49.04 ± 4.62 MPa. Preliminary cytocompatibility was confirmed by the MTT assay using MG63 cells. Following implantation in a rat calvarial defect model, robust osteogenesis and de novo bone formation were observed over an 8-week period.

A rat critical-sized calvarial defect model was also employed to evaluate the osteoinductive potential of PHB/HA hybrid scaffolds filled with alginate (ALG) hydrogel containing MSCs [130]. These constructs were prepared using a two-stage salt-leaching process combined with a 3D-printed mold. After promising in vitro findings, in vivo micro-CT and histological analyses demonstrated nearly complete defect regeneration after 28 days. Notably, the MSC-loaded scaffolds induced 3.6-fold greater bone formation at 22–28 days compared with acellular controls.

To the best of our knowledge, computer-aided wet-spinning (CAWS) was first applied by Pecorini et al. to fabricate PHBV-based composite scaffolds containing a dispersed PLGA phase with microfibrillar morphology embedded in a continuous PHBV matrix [129]. Increasing the PLGA fraction in the starting solution led to larger pore size, improved wettability, and enhanced thermal stability. In vitro tests with murine pre-osteoblasts confirmed scaffold biocompatibility and osteoconductivity, with higher proliferation rates observed for PLGA-rich formulations. The same group developed CAWS-derived PHBV/PLGA scaffolds loaded with HA (up to 15 wt%) [124]. HA incorporation increased compressive stiffness to values comparable with trabecular bone and significantly enhanced MC3T3-E1 cell viability and mineralized extracellular matrix deposition relative to the unloaded controls. One year later, Pecorini et al. further optimized this platform by incorporating TCP into PHBV/PLGA scaffolds [123]. In this system, PHBV blended with 30 wt% PLGA was processed via CAWS and loaded with up to 15 wt% β-TCP, yielding scaffolds with fully interconnected porosity. A ceramic loading of 5 wt% significantly increased the compressive modulus and improved the viability of human MSCs. Overall, the authors concluded that combining PLGA blending with β-TCP reinforcement markedly improved the processability, biomechanical performance, and bioactivity of PHBV scaffolds.

## 7. Discussion

### 7.1. Degradation for BTE

Polymers for BTE should combine biocompatibility with controlled degradability, ideally enabling their gradual resorption once structural and biological functions are fulfilled. Degradation is driven by macromolecular chain cleavage and influenced by local conditions, including hydrolytic and oxidative processes, the latter being mediated by reactive species such as hydrogen peroxide and hypochlorite released by inflammatory cells. Accordingly, the design of biodegradable bone alloplasts must ensure not only predictable degradation behavior but also the formation of biocompatible, metabolizable byproducts that do not accumulate or induce adverse local responses [35].

Another critical challenge for intraosseous polymeric scaffolds is their tendency to swell in aqueous environments, leading to volumetric expansion that may compromise mechanical stability at the implantation site. This effect is particularly relevant under physiological conditions associated with implantation, where local inflammation can reduce pH to ~6 or lower [135], and even to values as low as pH 3.0 during active bone remodeling [136]. Such acidic microenvironments can exacerbate polymer swelling, induce local tissue irritation, and generate internal stresses within composite systems. Moreover, pH-dependent degradation kinetics must be considered, as acidic conditions may alter hydrolytic degradation rates and mechanisms, ultimately affecting material resorption and performance [137].

**Figure 2:**
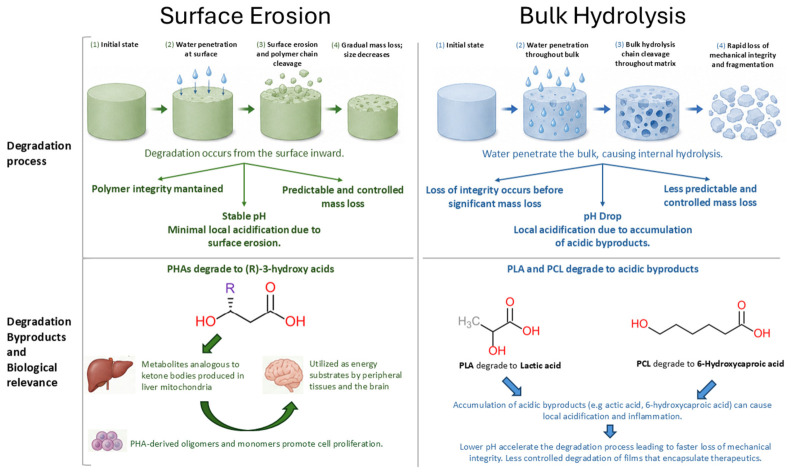
Description of the different degradation processes of PHAs by surface erosion and PCL/PLA by bulk hydrolysis.

In contrast to polyesters such as PGA and PLA, which can induce local acidification during bulk hydrolytic degradation, PHAs exhibit a more stable pH profile. This behavior is primarily attributed to their degradation mechanism, which proceeds predominantly via surface erosion rather than bulk hydrolysis (Figure 2), as described by Gould et al. [138], further confirmed by Shishatskaya et al. [139], and reviewed by Żur-Pińska et al. [140], who highlighted the beneficial effect of the layer-by-layer erosion process on the mechanical performance of the material, as it allows the core structure to remain relatively intact during degradation. Such a mechanism is also particularly advantageous for drug delivery applications, as it enables more predictable and controlled release kinetics of encapsulated therapeutics [141,142,143]. PHA degradation yields (R)-3-hydroxy acids, which are naturally occurring metabolites derived from the β-oxidation pathway [144]. These byproducts are readily assimilated and may contribute to local metabolic activity, potentially supporting tissue regeneration processes [145]. Notably, they include metabolites analogous to ketone bodies produced in liver mitochondria and utilized as energy substrates by peripheral tissues and the brain [36]. Moreover, PHA-derived oligomers and monomers have been reported to promote cell proliferation, further supporting their suitability for biomedical applications [144].

### 7.2. Mechanical Properties for BTE

The physicochemical properties of PHAs can be finely tuned through monomer composition, as they incorporate a wide range of structural units [146]. While PHB remains the most extensively studied PHA, the copolymer PHBV has gained significant attention due to its tunable mechanical properties, which depend on the ratio of 3-hydroxybutyrate (HB) to 3-hydroxyvalerate (HV) units. In particular, Shishatskaya et al. reported that 3D-printed PHBV scaffolds can achieve a Young’s modulus of 241.34 ± 7.62 MPa and a compressive strength of 22.43 ± 1.89 MPa [127], fully supporting their usage as bone substitutes, even in load bearing applications. Moreover, copolymer composition may also affect the degradation kinetics, allowing for the modulation of implant stability and resorption to better match the tissue regeneration rates [147].

Nevertheless, most of the studies reviewed here focused on enhancing the mechanical and biological performance of PHAs through the incorporation of inorganic and hybrid fillers. Among these, HA [112,116,117,124] remains the most widely investigated biomimetic phase, alongside β-TCP [18,123]. Additional fillers, including silicate-substituted HA (SiHA) [111], BGs [133], MWCNTs [92], GO [98,110], and alumina nanowires [99,108], have also been explored. More unconventional reinforcements, such as diatom shells (DS) [106] and zein [95], have also been tested. Very recently, barium titanate has been employed to impart piezoelectric stimulation through low-intensity pulsed ultrasound [148].

### 7.3. Functionalization for BTE

PHAs are highly versatile polymers that can be processed into fibers, films, and porous scaffolds using techniques such as electrospinning and AM, enabling precise control over the architecture and functional properties for BTE. This versatility is exemplified by the bi-layered tubular construct with multiple biomimetic interfaces designed to reproduce the hierarchical osteon structure, as reported by Sriram et al. [94]. Similarly, advanced multifunctional designs have been achieved with 3D-printed PHB scaffolds functionalized with dextran and charged Mg-doped whitlockite nanoparticles, further integrated with an angiogenic drug delivery platform for sildenafil release [122].

PHAs offer extensive opportunities for chemical and surface modification [149], enabling their use as platforms for protein immobilization [150] and biofunctionalization [151]. Functionalization strategies—including hydroxylation, carboxylation, and chlorination—enable the modulation of surface chemistry and bulk properties [152]. Although inherently hydrophobic [153], PHA surface wettability can be improved via approaches such as carboxyl ion implantation [154] or plasma treatment [100,114].

Furthermore, degraded PHAs may exert bacteriostatic effects, and both scl- and mcl-PHAs have been proposed for infection control applications [155]. The antibacterial effect of PLA/PHBV-based scaffolds was first described by Huang et al., who demonstrated that the incorporation of PHBV significantly enhanced the antibacterial activity exhibited by PLA alone [156]. This antimicrobial activity was further confirmed by Ma et al., who showed that PHB oligomers exerted effective antibacterial activity and promoted wound healing in infected nude mice [157]. Subsequently, Zhang et al. demonstrated a synergistic antimicrobial effect between polyethylene glycol (PEG) and PHB [158]. PHA-derived materials have also been used to fabricate biodegradable implantable rods acting as local antibiotic carriers for the treatment of chronic osteomyelitis [159]. Hybrid nanocomposites of PHB-HV with nanohydroxyapatite (nHA), fullerene (C60), and vancomycin (VC) were also tested as bone filler and local antibiotic therapy, as an alternative method to treat bone infections, showing encouraging results for the treatment of osteomyelitis through the controlled release of VC and also as a support for tissue regeneration [160].

At the application level, increasing attention is being directed toward “smart” PHA-based composites capable of responding to physiological stimuli or delivering therapeutic agents in a controlled manner. Drug-loaded systems, as demonstrated in the seminal work of Li et al. [132], highlight the potential of multifunctional scaffolds that simultaneously promote bone regeneration and provide localized anti-infective therapy. Such approaches are consistent with the broader transition toward personalized and functionally integrated implants. Equally relevant is the incorporation of pro-angiogenic cues, as reported in previous studies [122,131]. Indeed, robust osteogenesis and de novo bone formation are physiologically coupled with vascularization [161]. This aspect is particularly critical for overcoming one of the major limitations of BTE, namely the regeneration of large bone defects, where rapid and functional neovascularization is essential for tissue survival and integration. Poly(3-hydroxybutyrate-co-3-hydroxyvalerate-co-3-hydroxyhexanoate) (PBVHx) nanoparticles with soybean lecithin-modified BMP4 have been tested as a novel long-acting BMP4 delivery system, showing their osteo-differentiative potential in hBMSCs [162].

### 7.4. Barriers to the Clinical Use of PHA Scaffolds

Despite the substantial volume of research on PHAs generated to date, only a limited number of studies have provided in vivo validation, and none have progressed to clinical application. High production costs and the lack of supportive industrial and regulatory frameworks have likely contributed to the limited diffusion of PHAs thus far [163]. Several challenges and opportunities therefore remain for successful clinical translation. A major limitation of PHAs is their thermal instability. For example, PHB exhibits a melting temperature around 180 °C, while thermal degradation may begin above 220 °C, resulting in a narrow processing window. Thermal degradation significantly compromises the structural fidelity required for the fabrication of larger scaffolds, which depend on the deposition of multiple stacked layers. Under these conditions, the extruded struts may collapse before crystallization occurs, thereby limiting achievable and reproducible build heights. Consequently, the maximum clinically relevant scaffold size attainable through extrusion-based 3D printing is generally restricted to a few centimeters. Moreover, the relatively slow crystallization kinetics of PHAs can promote warping phenomena and loss of the predefined pore architecture during cooling. The development of novel blends, copolymers, and mcl-PHA-based systems could significantly expand processability and application range [164]. An emerging strategy involves extremophilic microorganisms, which may combine the compositional control of pure cultures with non-sterile operation, thereby reducing operational costs and simplifying downstream recovery [165]. In parallel, future efforts should target improved biosynthesis efficiency and cost reduction through feedstock valorization and bioprocess optimization [166,167]. Across all production routes, however, strict control of polymer composition, molecular weight, and purity remains essential. Indeed, medical-grade PHAs require efficient purification to remove residual pyrogens, including endotoxins and exotoxins derived from microbial biomass. The downstream processing required to convert microbial biomass into highly pure, sterile, medical-grade biopolymers represents a substantial barrier to clinical translation and commercialization. In addition, scaling up production while maintaining strict batch-to-batch consistency, a crucial requirement for regulatory compliance, significantly increases the manufacturing complexity and costs. Although PHAs exhibit excellent biocompatibility, their intrinsic mechanical properties, such as brittleness in highly crystalline formulations and hydrophobicity, often require modification or combination with reinforcing materials, including bioglass or hydroxyapatite, in order to satisfy the mechanical demands of load-bearing bone applications. The optimization and validation of these hybrid composite systems inevitably prolong both the research and development phase and the clinical trial timeline.

## 8. Conclusions

Collaboration among academic researchers, industry, and clinical stakeholders will be essential to accelerate the translation of PHA-based BTE from the laboratory to clinical settings, ensuring that these innovative materials meet the regulatory standards and are successfully implemented. Overall, PHAs hold significant promise for the future of regenerative medicine. Their combination of tunable properties, bioactivity, biodegradability, and sustainability positions them as attractive candidates to address current clinical limitations and the growing demand for advanced, environmentally responsible biomaterials.

## Figures and Tables

**Table 1:** Substrates for PHA production. PCM: pure-culture microorganisms. MMC: mixed microbial cultures.

Microbial System	Substrate	References
PCM	Whey	Kim et al., Koller et al., Yellore et al. [45,46,47]
PCM	Starch	Kim et al., Halami et al. [45,48]
PCM	Vegetable oils	Obruca et al., Taniguchi et al. [49,50]
PCM	Bagasse and cellulosic materials	Alva Munoz et al., Silva et al., Yu et al. [51,52,53]
PCM	Molasses	Gouda et al., Jiang et al., Liu et al. [54,55,56]
PCM	Glycerol	Koller et al., Cavalheiro et al. [46,57]
PCM	Fermented food waste	Du et al. [58]
MMC	Food waste	Rhu et al. [59]
MMC	Tomato cannery wastewater	Liu et al. [60]
MMC	Olive oil mill wastewater	Dionisi et al., Villano et al. [61,62]
MMC	Domestic wastewater	Beccari et al. [63]
MMC	Fermented molasses	Albuquerque et al., Bengtsson et al., Dias et al. [64,65,66,67,68]
MMC	Paper mill wastewater	Bengtsson et al. [69]
MMC	Municipal wastewater	Morgan-Sagastume et al. [70]

**Table 2:** Electrospun materials.

Authors	Scaffold Material	Mechanical Features	Level of Evidence	
			In Vitro	In Vivo
Wu et al. [92]	Poly (3-hydroxybutyric acid-co-4-hydroxybutyric acid) (P34HB) and polyvinyl alcohol (PVA) were employed as matrix materials, with multi-walled carbon nanotubes (MWCNTs)	Pure PVA scaffolds exhibited superior Young’s modulus (7.63 ± 0.99 MPa) compared to pure P34HB (0.43 ± 0.03 MPa) and hybrid PVA-P34HB scaffolds (2.74 ± 0.22 MPa). This enhancement correlated directly with MWCNT concentration	Human BMSC viability, proliferation, osteogenic differentiation	White rabbits with femoral condyle loosening. Hybrid electrospun scaffolds incorporating 1% MWCNTs demonstrated superior osteogenic potential and enhanced pull-out resistance
Sriram et al. [94]	Polyhydroxybutyrate (PHB) + gelatin fibers (PG) to fabricate mineralized fibers (mPG). A bi-layered tubular construct that had PG in the outer layer and 7%PHB/0.5%Polypyrrole fibers (PPy) in the inner layer	Not assessed	Mouse BMSC viability, proliferation, osteogenic differentiation	Not assessed
Ghasemi et al. [95]	Zein/poly(hydroxybutyrate) (PHB) electrospun scaffolds	Zein increased tensile stress (from 2.94 ± 0.23 MPa of PHB only to 4.49 ± 0.25 MPa) and elongation at break (from 11.8 ± 0.8% of PHB only to 82.6 ± 2.3%)	Human MG-63 cell line used to evaluate adhesion, viability, osteogenic differentiation	Not assessed
Zonari et al. [96]	PHB and PHB-co-3-hydroxyvalerate (PHB-HV)	Not assessed	Human adipose tissue-derived stem cells (hASCs) to test viability, adhesion, morphology and their endothelial differentiation	Nude mouse model of the critical-sized calvarial defect showed hASCs cultured on PHB-HV scaffolds were ineffective to promote bone regeneration it improved scaffold vascularization
Li et al. [97]	P34HB + ciprofloxacin (CIP) + dimethyloxalylglycine (DMOG)	Increased tensile stress to 4.08 ± 0.18 MPa and elongation break to 354.8 ± 18.4%	Mouse L929 cells used to evaluate cell proliferation, spreading and migration	Mouse full thickness excisional wound model showed a complete skin regeneration and hair follicles with increased re-epithelialization, collagen formation, angiogenesis and remodeling
Motiee et al. [98]	Poly-3 hydroxybutyrate-chitosan (PC) scaffolds + graphene oxide (GO)	Increasing percentage of GO improved tensile strength (from 0.84 ± 1.18 MPa to 8.175 ± 0.59 MPa with 0.5% GO), Young’s modulus of about 4.5-fold and elongation of about 3-fold	Human MG-63 cell line used to evaluate cellular adhesion and viability	Not assessed
Ghafari et al. [99]	Polyhydroxybutyrate-keratin (PHB-K) + alumina nanowire	Alumina nanowire increased tensile strength (from 2.31 ± 0.11 to 6.72 ± 0.12 MPa), Young’s modulus (from 101.32 ± 17 MPa to 168.45 ± 4 MPa) and elongation (from 3.84 ± 0.02% to 23.02 ± 0.25%)	Rat adipose tissue-derived mesenchymal stem cells (MSCs) used to test viability, adhesion, morphology, osteogenic differentiation	Not assessed
Mohammadalipour et al. [100]	PHB nanofibers with oxygen/argon plasma	Plasma surface modification of PHB nano fibers reduced the scaffolds’ tensile modulus (around 1.4-fold, reaching 99.85 ± 23.87 MPa) and strength (around 1.3-fold reaching 2.89 ± 0.44 MPa)	Human MG-63 cell line used to evaluate adhesion, viability, osteogenic differentiation	Not assessed
Asl et al. [93]	PHB-starch-multiwalled carbon nanotubes (MWCNTs)	MWCNT increased tensile strength (24.37 ± 0.22 MPa)	Human MG-63 cell line used to evaluate adhesion, viability, osteogenic differentiation	Not assessed
Tahmasebi et al. [101]	Aloe vera-derived gel-blended poly(3-hydroxybutyrate-co-3-hydroxyvalerate) (PHBV)	Strain and elongation at break of PHBV scaffold, after coating with gel, increased (from 67.38 ± 5.1% to 77.28 ± 2.89%), while PHBV Young’s modulus decreased (from 1.6 ± 0.35 to 1.2 ± 0.45 MPa)	Human-induced pluripotent stem cells (iPSC) tested for cell adhesion, viability and osteodifferentiation	Not assessed
Wang et al. [102]	Poly(l-lactide) (PLLA) + poly(3-hydroxybutyrate-co-3-hydroxyvalerate) (PHBV)	The Young’s modulus increased for PLLA-PHBV blends (from 41.5 ± 3.3 of PLLA to 73.5 ± 3.2 MPa)	Mouse bone mesenchymal stem cells tested for osteodifferentiation	Not assessed
Wang et al. [103]	Poly(3-hydroxybutyrate-co-4-hydroxybutyrate) (P34HB)/octacalcium phosphate (OCP) (P34HB)/(OCP)	OCP enhanced tensile strength of (P34HB) reaching 2.73 ± 0.03 MPa	Rat mesenchymal stem cells tested for cell adhesion, viability and osteodifferentiation	Bone repair of a calvarial bone defect in a rat model
Kara et al. [104]	Fish scale/poly(3-hydroxybutyrate-co-3-hydroxyvalerate) (PHBV)	Incorporation of the fish scales increased about 2.5-fold compressive modulus of the scaffold (reaching 2.5 MPa)	Human MG-63 cell line used to evaluate cell cytotoxicity, proliferation and osteogenic differentiation	Not assessed
Ang et al. [105]	Surface coating of poly(3-hydroxybutyrate-co-3-hydroxyhexanoate) (PHBHHx) with silk fibroin (SF)	Not assessed	Human umbilical cord-derived mesenchymal stem cells (hUC-MSCs) used to study morphology, growth and proliferation, and osteogenic differentiation	Not assessed
Dalgic et al. [106]	Poly(hydroxybutyrate-co-hydroxyvalerate)/poly(ε-caprolactone) (PHBV/PCL) + and DS incorporated pullulan (PUL) fibers	Increase in Young’s modulus (around 5.4-fold, reaching 515.2 ± 91.9 kPa) and elastic modulus and decrease in tensile strength of PHBV/PCL/DS scaffold (reaching 19.1 ± 1.4 kPa)	Human primary sarcoma cell line (Saos-2) used to investigate osteocompatibility	Not assessed
Fu et al. [107]	Poly3-hydroxybutyric acid 4hydroxybutyrate4-hydroxybutyrate (P34HB)	Good values for elongation (26.9%) and Young’s modulus (58.929 MPa)	Murine bone marrow mesenchymal stem cells (BMSCs) tested for cellular morphology and viability	Bone repair of a rat calvarial defect
Toloue et al. [108]	Alumina nanowires are added to (polyhydroxybutyrate-chitosan (HB-CTS)	Al_2_O_3_ increased >10 fold (reaching 11.18 ± 1.24 MPa) the tensile strength compared to PHB-CTS	Human MG-63 cell line used to evaluate morphology, viability and osteogenic differentiation	Not assessed
Kouhi et al. [109]	Poly (hydroxybutyrate-co-3-hydroxyvalerate) (PHBV)/fibrinogen (FG)/bredigite (BR) membranes	Young’s modulus and strength of the PHBV membrane reduced upon blending with FG (83.3 ± 15.5 MPa and 3.68 ± 1.1 MPa) and increased by further incorporation of BR nanoparticles (121 ± 14.4 MPa and 5.22 ± 0.7 MPa)	Human fetal osteoblast cells (hFOB) tested for viability and osteoblast differentiation	Not assessed
Zhou et al. [110]	Poly(3-hydroxybutyrate-co-4-hydroxybutyrate) (P34HB)/Graphene Oxide (GO)	Scaffolds with 2 mg/mL GO were improved by 87% in tensile strength (reaching 2.02 ± 0.24) and by 161% in Young’s modulus (reaching 85.11 ± 2.86 MPa) were increased by GO	Rat bone marrow mesenchymal stem cells (BMSCs) tested for adhesion, viability, proliferation, and osteogenic differentiation	P34HB/GO was superior to P34HB in bone regeneration in critical-sized calvarial defects of rats
Gorodzha et al. [111]	Poly(3-hydroxybutyrate-co-3-hydroxyvalerate) (PHBV) and a combined with silicate containing hydroxyapatite (PHBV-SiHA)	Not assessed	Human bone marrow mesenchymal stem cells (BMSCs) tested for cellular adhesion, morphology, viability and osteogenic differentiation	Not assessed
Chen et al. [112]	Hydroxyapatite nanoparticle (nHA)/poly-hydroxybutyrate (PHB)	Tensile strength at break, tensile strain at break and elastic modulus increased reaching 3.99 ± 0.57 MPa, 3.46 ± 0.93%, and 798.25 ± 120.07 MPa respectively with nHA	Rabbit bone marrow mesenchymal stem cells (BMSCs) tested for adhesion, proliferation and osteogenic differentiation	The scaffold was implanted in BALB/c nude mice, which showed osteoid tissue formation and blood vessel ingrowth was detected into the graft
Zhang et al. [113]	Poly-3-hydroxybutyrate-co-3-hydroxyvalerate (PHBV)/polyaspartic acid (PAA) + nano-hydroxyapatite (nHA)	PHBV/PAA-nHA scaffolds are suitable for bone tissue reparation having a tensile strain of 15.78% which is higher than the one of natural bone (3–5%) nHA improved tensile strength	Human fetal osteoblast (hFOB) tested for cellular adhesion, proliferation and osteogenic differentiation	Not assessed
Unalan et al. [114]	Plasma treated-poly (3-hydroxybutyrate-co-3-hydroxyvalerate) (PHBV) and silk fibroin (SF)-PHBV	Not assessed	SaOs-2osteoblast-like human osteosarcoma cells tested for cellular morphology, viability and osteogenic differentiation	Not assessed
Paşcu et al. [115]	Polyhydroxybutyrate-polyhydroxyvalerate(PHBV)/nanohydroxyapatite (nHA)/silk fibroin (SF)	Compressive and tensile mechanical properties improved with nHA and SF (Young ‘s modulus ranging between ~1.5–2.8 MPa)	Mouse calvarian osteoblast cells (MC3T3-E1), tested for cellular morphology, adhesion, proliferation and osteogenic differentiation	Not assessed
Zhang et al. [116]	Poly-3-hydroxybutyrate-co-3-hydroxyvalerate (PHBV) + poly-(α, β)-DL-aspartic acid	Not assessed	Human fetal osteoblasts (hFOB) were cultured to test cell proliferation and osteodifferentiation	Not assessed
Ramier et al. [117]	Poly(3-hydroxybutyrate) (PHB) + nanohydroxyapatite (nHA)	n-HA increased the elastic modulus by 67% (reaching 397 ± 107 MPa) and tensile strength at break by 51% (reaching 16.16 ± 0.86 MPa)	Human mesenchymal stem cells (hMSC) to test cellular adhesion, morphology and proliferation	Not assessed
Paşcu et al. [118]	Polyhydroxybutyrate-co-(3-hydroxyvalerate) with 2% valerate fraction (PHBV), nano-hydroxyapatite (nHAp), and Bombyx mori silk fibroin essence (SF)	n-HA and SF at 2% increased Young’s modulus from 0.7 kPa (±0.33 kPa) of PHBV only to 1.4 kPa (±0.54 kPa)	Human osteoblasts tested for cellular adhesion, morphology and viability	Not assessed

**Table 3:** 3D printed materials for BTE applications.

Authors	PHA	Type of Manufacturing	Mechanical Features	Level of Evidence		Notes
				In Vitro	In Vivo	
Nazar et al. [122]	Polyhydroxybutyrate (PHB), functionalized with dextran (Dex), charged Mg-doped whitlockite (WL) nanoparticles, coated with sildenafil-loaded Dex-Pluronic F127 nanofibers.	Extrusion-based 3D printer (RegenHU, Switzerland)	Compressive strength of 3.70 ± 0.33 MPa and an elastic modulus of 49.04 ± 4.62 MPa	MG63 cell line tested for viability, cell adhesion, ALP activity and calcium deposition	Angiogenic potential assessed through a chick chorioallantoic membrane (CAM) assay. Bone regeneration tested via rat calvarial defect model	Sildenafil, a phosphodiesterase-5 (PDE5) inhibitor, may improve angiogenesis and tissue repair through its vasodilatory effects
Pecorini et al. [123]	Poly(3-hydroxybutyrate-co-3-hydroxyvalerate) (PHBV) and PLGA loaded with β-TCP (up to 15 wt%)	Computer-aided wet-spinning (CAWS)	β-TCP at 5 wt% increased the compressive modulus reaching around 6 MPa	Human mesenchymal stem/stromal cells tested for viability and osteodifferentiation	Not assessed	Not assessed
Pecorini et al. [124]	Poly(3-hydroxybutyrate-co-3-hydroxyvalerate) (PHBV) and PLGA loaded with HA (up to 15 wt%)	Computer-aided wet-spinning (CAWS)	HA loading increased scaffold compressive stiffness (average compressive modulus from 1.3 MPa of PHBV scaffold to 4.6 MPa for 5% and 9.3 MPa for 10% and 15% PHBV/HA loaded scaffolds)	Viability of murine pre-osteoblastic cell line MC3T3-E1 and their mineralization capability	Not assessed	Not assessed
Dominguez-Candela et al. [125]	Polyhydroxybutyrate (PHB) and PLA functionalized with poly(ethylene) with glicidyl metacrylate (EGM) and methyl acrylate-co-glycidyl methacrylate (EMAG); poly-(styrene-co-maleic anhydride) copolymer (Xibond); and bio-based epoxidized linseed oil (ELO)	Material extrusion (MEX) additive manufacturing (AM) technology	Compression stress was 11–13 MPa	Human fetal osteoblastic (hFOB) cells tested for viability and cellular proliferation	Not assessed	Not assessed
Marcello et al. [126]	Poly(3-hydroxyoctanoate-co-hydroxydecanoate-co-hydroxydodecanoate) (P(3HO-co-3HD-co-3HDD) + PHAs containing thioester groups in their side chains (i.e., PHACOS) or charged with HA doped with selenium and strontium ions (Se-Sr-HA)	Melt-extrusion 3D printing	Not assessed	MC3T3-E1 cells used to test viability and osteodifferentiation	Not assessed	Antibacterial features tested on *Staphylococcus aureus* 6538P and *Escherichia coli* 8739
Shishatskaya et al. [127]	Poly(3-hydroxybutyrate-co-3-hydroxyvalerate) (PHBV)	FDM	Young’s modulus and compressive stress tensile strength increased, according to the direction of load application (parallel or perpendicular to the layers of the scaffold: Young’s modulus of 207.52 ± 19.12 and 241.34 ± 7.62 MPa and compressive stress tensile strength of 19.45 ± 2.10 and 22.43 ± 1.89 MPa)	NIH 3T3 fibroblasts tested for viability and metabolic activity	The diaphyseal zone of the femur in domestic Landrace pigs up to 150 days of observation	Not assessed
Zhang et al. [128]	Polyhydroxyalkanoates/β-tricalcium phosphate (PHA/β-TCP) enriched with copper-containing carboxymethyl chitosan/alginate (CA/Cu) hydrogels	FDM	Not assessed	MC3T3-E1 cell tested for viability, adhesion and osteogenic differentiation	Accelerated bone repair of cranial defects in Sprague-Dawley rats	Methicillin-resistant *Staphylococcus aureus* (MRSA)
Ye et al. [18]	Ecomann^®^ Bioresin EM10080 filled with β-TCP (up to 30 wt%)	FDM	The compressive strength and the shore hardness of the PHA/20%β-TCP scaffold were 36.7 MPa and 81.1 HD	MC3T3-E1 cells tested for proliferation, adhesion, and migration	Not assessed	Not assessed
Berger et al. [120]	Polyhydroxybutyrate (PHB) loaded with β-TCP	3D printing	Not assessed	Human mesenchymal stem cells (hMSCs) tested for viability and osteogenic differentiation	Not assessed	Not assessed
Pecorini et al. [129]	Poly(3-hydroxybutyrate-co-3-hydroxyvalerate) (PHBV) and poly(D,L-lactide-co-glycolide) (PLGA)	Computer-aided wet-spinning (CAWS)	PHBV/PLGA weight ratios of 50/50 r compressive modulus increased up to 3.4 ± 2.1 MPa when dry and 3.7 ± 1.1 MPa when wet	MC3T3-E1 cell tested for viability, adhesion, morphology and osteogenic differentiation	Not assessed	Not assessed
Volkov et al. [130]	Poly(3-hydroxybutyrate) (PHB) with HA filled with alginate (ALG) hydrogel	Two-stage salt leaching technique using a mold obtained by 3D printing	The Young’s and shear moduli of PHB + HA increased, reaching 34.0 ± 0.6 kPa and 85.6 ± 3.0 kPa respectively. The scaffolds PHB/HA/ALG increased Young’s modulus (178.5 ± 1.8 kPa)	Wistar rat f femur isolated mesenchymal stem cells used to test viability and osteodifferentiation	Critical-sized calvarial defect in rats filled with either PHB/HA/ALG/MSC scaffolds or PHB/HA/ALG scaffolds. The former overperforming the latter	Not assessed
Saska et al. [121]	Poly(3-hydroxybutyrate) (PHB) functionalized with osteogenic growth peptide (OGP) and its C-terminal sequence OGP(10–14)	Selective laser sintering (SLS)	Compressive modulus showed good value (4.91 ± 0.30 MPa)	Rat bone marrow stem cells tested for viability and calcium precipitation	Not assessed	Not assessed
Min et al. [131]	Poly(3-hydroxybutyrate-co-3-hydroxyhexanoate) (PHBHHx) charged with mesoporous bioactive glasses and releasing dimethyloxallyl glycine (DMOG)	3D-bioplotter system (EnvisionTEC GmbH 3D Bioplotter™)	Not assessed	Human bone marrow stromal cell (human BMSC) adhesion, viability, proliferation, osteogenic differentiation and angiogenic-relative gene expressions	Calvarial defects were created in Sprague-Dawley rats. DMOG could be released in a sustained manner over 4 weeks enhancing the angiogenesis and osteogenesis in the defects	Not assessed
Li et al. [132]	Poly(3-hydroxybutyrate-co-3-hydroxyhexanoate) (PHBHHx)	3D-bioplotter system (EnvisionTEC GmbH 3D Bioplotter™)	Not assessed	Not assessed	New Zealand rabbits’ femur defect model resulted repaired in the drug-loaded composite scaffold (DLCS) group and the β-TCP group	Not assessed
Yang et al. [133]	Poly(3-hydroxybutyrate-co-3-hydroxyhexanoate) (PHBHHx) either coated with mesoporous bioactive glass (MBG) or not	3D printing	Not assessed	Human mesenchymal stem cells (hMSCs) viability, adhesion and osteogenic differentiation	Not assessed	Not assessed

## Data Availability

No new data were created or analyzed in this study. Data sharing is not applicable to this article.

## Abbreviations

The following abbreviations are used in this manuscript:

ALG	Alginate
ALP	Alkaline phosphatase
AM	Additive manufacturing
BG	Bioglass
BMSC	Bone marrow stromal cell
BR	Bredigite
BTE	Bone tissue engineering
CAM	Chorioallantoic membrane
CAWS	Computer-aided wet-spinning
Dex	Dextran
DLCS	Drug-loaded compo-site scaffold
DMOG	Dimethyloxallyl glycine
DSDiatom shellEGM	Poly(ethylene) glycidyl methacrylate
ELO	Epoxidized linseed oil
EMAG	Poly(ethylene) methyl acrylate-co-glycidyl methacrylate
FDM	Fused deposition modeling
FG	Fibrinogen
GBR	Guided bone regeneration
GO	Graphene oxide
HA	Hydroxyapatite
HB	3-hydroxybutyrate
hFOB	Human fetal osteoblast
HIF-1α	Hypoxia-inducible factor-1α
hUC-MSC	Human umbilical cord-derived mesenchymal stem cell
HV	3-hydroxyvalerate
iPSC	Induced pluripotent stem cell
MBG	Mesoporous bioactive glass
MC3T3-E1	Mouse calvarian osteoblast cells
mcl-PHA	Medium-chain-length PHA
MEX	Material extrusion
MMC	Mixed microbial culture
mPG	Mineralized fibers
MRSA	Methicillin-resistant *Staphylococcus aureus*
MSC	Mesenchymal stromal cell
MWCNT	Multi-walled carbon nanotube
nHA	Hydroxyapatite nanoparticle
OCP	Octacalcium phosphate
OGP	Osteogenic growth peptide
P(3HB-co-3HV)	Poly(3-hydroxybutyrrate-co-3-hydroxyvalerate)
P(3HO-co-3HD-co-3HDD)	Poly(3-hydroxyoctanoate-co-hydroxydecanoate-co-hydroxydodecanoate)
P(4HB)	Poly(4-hydroxybutyrate)
P34HB	Poly(3-hydroxybutyric acid-co-4-hydroxybutyric acid)
PAA	Polyaspartic acid
PBVHx	Poly(3-hydroxybutyrate-co-3-hydroxyvalerate-co-3-hydroxyhexanoate)
PC	Poly-3 hydroxybutyrate-chitosan
PCL	Polycaprolactone
PCM	Pure-culture microorganism
PDEP	Phosphodiesterase-5
PEG	Poly(ethylene glycol)
PG	Gelatin fibers
PHA	Polyhydroxyalkanoate
PHB	Poly(3-hydroxybutyrate)
PHB-CTS	Polyhydroxybutyrate-chitosan
PHBHHx	Poly(3-hydroxybutyrate-co-3-hydroxyhexanoate)
PHB-K	Polyhydroxybutyrate-keratin
PHBV	Poly(3-hydroxybutyrate-co-3-hydroxyvalerate)
PHHx	Poly(3-hydroxyhexanoate)
PHO	Poly(3-hydroxyoctanoate)
PHV	Poly(3-hydroxyvalerate)
PLA	Polylactic acid
PLGA	Poly(lactide-co-glycolide)
PLLA	Poly(l-lactide)
PPy	Polypyrrole fibers
PUL	Pullulan
PVA	Polyvinyl alcohol
Saos-2	Human primary sarcoma cell line
scl-PHA	Short-chain-length PHA
SEM	Scanning electron microscope
SF	Silk fibroin
SLS	Selective laser sintering
TCP	Tricalcium phosphate
WL	Whitlockite
Xibond	Poly(styrene-co-maleic anhydride)
β-TCP	β-Tricalcium phosphate
